# Compression of the Porta Hepatis in a Patient With Hepatic Cysts Secondary to Autosomal Dominant Polycystic Kidney Disease: A Case Report

**DOI:** 10.7759/cureus.111667

**Published:** 2026-06-28

**Authors:** Yasasvhinie Santharam, Kabeer Ali, Anvit D Reddy, Aleem A Ali, Vishal Jaikaransingh

**Affiliations:** 1 Internal Medicine, University of Florida College of Medicine - Jacksonville, Jacksonville, USA; 2 Gastroenterology, University of Florida College of Medicine - Jacksonville, Jacksonville, USA; 3 Nephrology, University of Florida College of Medicine - Jacksonville, Jacksonville, USA

**Keywords:** autosomal dominant polycystic kidney disease (adpkd), compression of porta hepatis, extra-renal manifestation of adpkd, hepatic cyst, hepatic vasculature

## Abstract

Autosomal dominant polycystic kidney disease (ADPKD) is a genetic disorder characterized by cyst formation in the kidneys and other organs: most commonly the liver, pancreas, and seminal vesicles. Hepatic cysts are one of the most common extrarenal manifestations of ADPKD, but generally remain asymptomatic. Rarely, they can cause mass effect leading to biliary obstruction, portal hypertension, or compression of adjacent vascular structures.

We present the case of a 67-year-old woman with ADPKD, large hepatic cysts causing compression of the porta hepatis, and biliary strictures requiring stenting, in the absence of another accompanying liver disease.

## Introduction

ADPKD has an estimated prevalence in the United States of approximately 42.6 per 100,000 persons [1]. It generally results from mutations in two genes: PKD1 (85%-90% of the time) and PKD2 (10%-15% of the time), with PKD2 patients having a later onset of end-stage renal disease (ESRD) [2]. Although named as a kidney disease, it is a systemic disorder. Extrarenal manifestations include polycystic liver disease (PCLD/PLD), valvular heart disease, intracranial aneurysms, diverticulosis, and pancreatic cysts [2]. Importantly, PCLD in ADPKD must be differentiated from isolated PCLD, which occurs in patients who do not have concurrent renal disease. The incidence of clinical sequelae in isolated PLD versus PLD secondary to ADPKD has not yet been well established, with existing data being extrapolated to both conditions. This distinction is clinically important as isolated PLD may have a different natural history and management approach. Per the 2024 American College of Gastroenterology (ACG) clinical guidelines on focal liver lesions, up to 60%-80% of patients with ADPKD have hepatic cyst involvement [3]. Both the prevalence and average volume of hepatic cysts is higher in women, with an increase in incidence with age, pregnancies, and estrogen exposure [2].

## Case presentation

A 67-year-old female with a past medical history of hypertension, type 2 diabetes, no prior estrogen use, and ESRD secondary to ADPKD presented to the hospital when routine outpatient laboratory tests were indicative of acute kidney injury and elevated liver enzymes. At the time, the patient was 14 years post living-related-donor kidney transplant due to ADPKD, having received thymoglobulin induction (7.5 mg/kg), and maintained on a well-tolerated regimen of tacrolimus, mycophenolate mofetil, and prednisone for immunosuppression. She reported feeling generally unwell with approximately three to four weeks of gradual scleral icterus, jaundice, dark urine, pale stools, and diffuse pruritus. The patient denied abdominal pain, nausea, vomiting, early satiety, or dyspnea.

In the Emergency Department, the patient was hypertensive but otherwise hemodynamically stable. Laboratory evaluation revealed hemoglobin 11.6 g/dL, total bilirubin 5.9 mg/dL, alkaline phosphatase (ALP) 1,426 IU/L, aspartate aminotransferase (AST) 343 IU/L, and alanine aminotransferase (ALT) 290 IU/L. Also noted in labs was an acute kidney injury superimposed on chronic kidney disease, with a creatinine of 4.09 mg/dL (baseline around 2.6 mg/dL) (Table 1).

**Table 1:** Laboratory values ALP: alkaline phosphatase; AST: aspartate aminotransferase; ALT: alanine aminotransferase

Laboratory Test (unit)	Patient’s Result	Normal Range
Creatinine (mg/dL)	4.09	Patient’s baseline Cr ≈ 2.6
Hemoglobin (g/dL)	11.6	12.0-15.5
Total Bilirubin (mg/dL)	5.9	0.2-1.0
ALP (IU/L)	1426	35-104
AST (IU/L)	343	14-33
ALT (IU/L)	290	10-42

**Figure 1:**
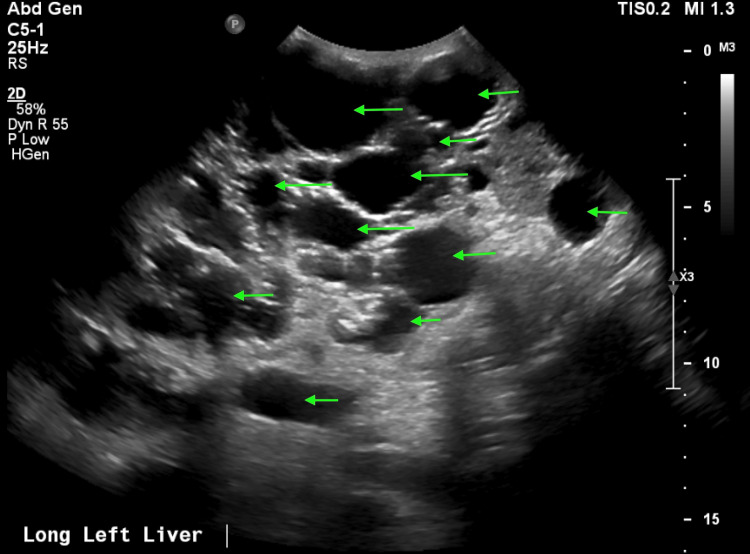
Ultrasound of the liver with several intrahepatic cysts (green arrows)

Ultrasound of the right upper quadrant showed multiple liver cysts consistent with those seen in polycystic kidney disease (PKD) (Figure 1), as well as an echogenic transplanted kidney visualized without hydronephrosis.

**Figure 2:**
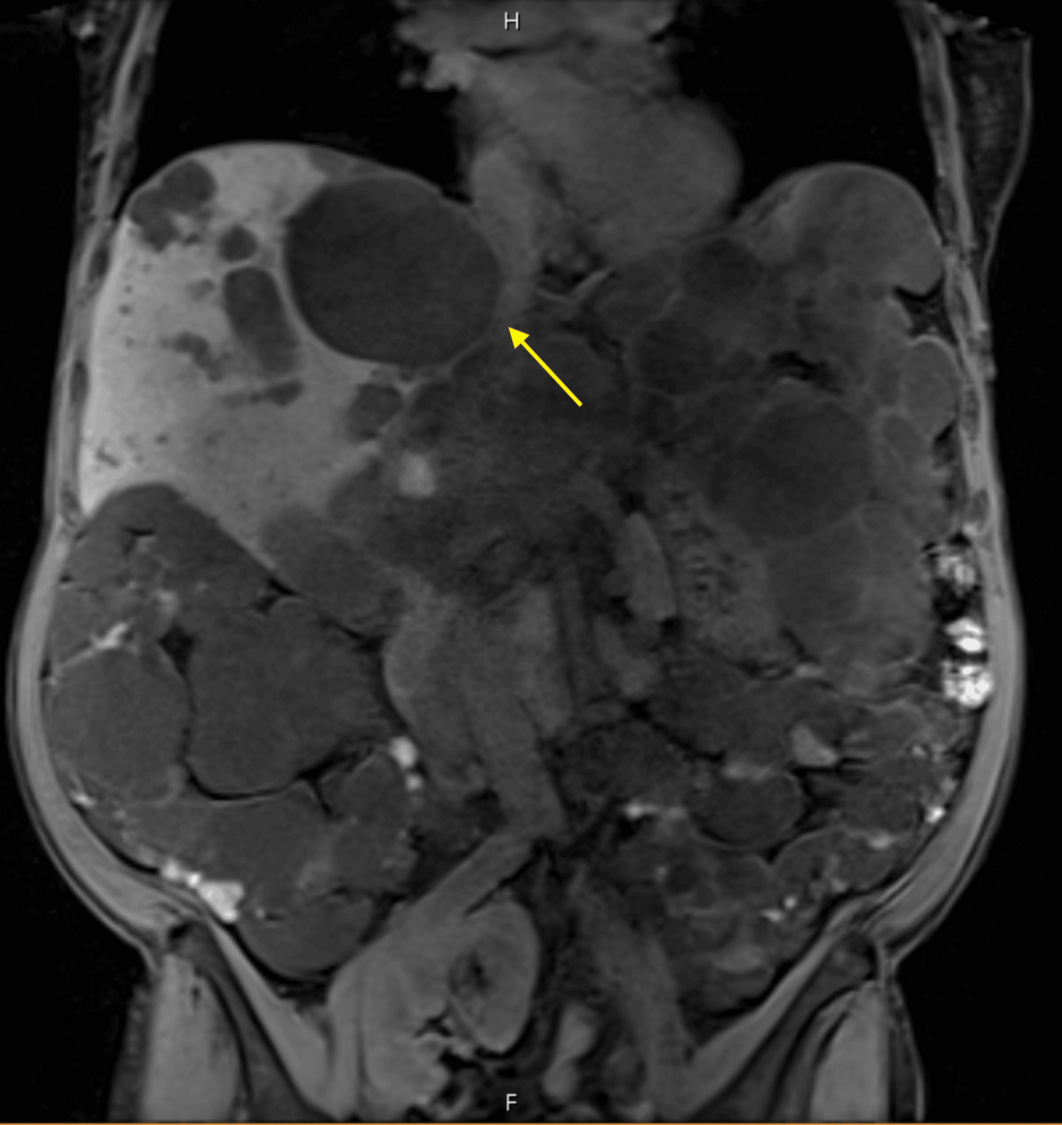
MRCP showing a large hepatic lesion compressing the porta hepatis (yellow arrow) MRCP: magnetic resonance cholangiopancreatography

Magnetic resonance cholangiopancreatography (MRCP) revealed evidence of intrahepatic ductal dilation and common hepatic and common bile duct narrowing due to mass effect from numerous hepatic cysts, including one large 6.7 cm by 6.9 cm hemorrhagic cyst that demonstrated external compression of the porta hepatis (Figure 2).

The patient was placed on a regimen of cholestyramine and diphenhydramine for her pruritus and transferred to a tertiary care center for transplant evaluation. Further evaluation of the biliary tree with endoscopic retrograde cholangiopancreatography (ERCP) was significant for multiple biliary strictures. A metal biliary stent was placed to relieve the biliary strictures, and a pancreatic duct stent was placed prophylactically due to difficult cannulation to minimize the risk of post-ERCP pancreatitis. One week later, the patient reported abdominal pain. A stat abdominal radiograph was obtained and revealed free air (pneumoperitoneum), concerning for bowel perforation. The patient was taken for an emergent repeat ERCP, which revealed migration of the biliary stent out of the bile duct with resultant duodenal wall perforation. Broad-spectrum antibiotics were initiated, and surgical consultation was obtained. Both initial stents were removed, and a fully covered metal stent was placed in the common bile duct. She was then started on hemodialysis for metabolic acidosis due to lactic acidosis from post-procedure sepsis, then became lightheaded and confused, with vitals showing a hypotensive blood pressure with mean arterial pressures less than 65, and labs revealing an acute drop in hemoglobin, leading to initiation of massive transfusion protocol. She was transferred to the surgical ICU and intubated for airway protection in the setting of acute hypoxic respiratory failure in the setting of large volume hemoperitoneum noted on bedside ultrasound.

The surgery team performed multiple exploratory laparotomies for duodenal perforation repair and fenestration of multiple liver and renal cysts. The patient was then worked up for the possibility of a simultaneous liver/kidney transplant due to ongoing need for renal replacement therapy and a MELD-Na score that had maintained around 29-32 throughout her hospitalization, indicating a >19% chance of three-month mortality. Given her ongoing critical illness as well as acute decompensation in the setting of a new post-surgical intra-abdominal abscess, she was considered a poor candidate for either transplant. Upon discussion with palliative care, the patient and her family decided to move forward with hospice and comfort measures prior to her passing.

## Discussion

PKD, particularly ADPKD, frequently manifests with hepatic cysts, which are the most common extrarenal manifestation of the disease [4]. The clinical importance of managing ADPKD patients who often progress to end-stage renal disease is underscored by a previous study demonstrating that chronic kidney disease contributes to 25.4% of sudden adult deaths, primarily from cardiovascular causes [5]. This highlights the need for comprehensive multidisciplinary care addressing both renal and extrarenal manifestations in this high-risk population.

The American Gastroenterological Association notes that liver enzymes and synthetic function are often preserved despite a heavy cyst burden, except in advanced cases [3]. However, some evidence contradicts the hypothesis that the biochemical hepatic burden of PKD is insignificant. One study by Judge et al. of over 1000 patients with PKD notes that the burden of biliary tract disease was more than serious liver disease, inguinal hernias, and cerebral aneurysms [6]. This case contributes to the growing body of evidence in this latter category, of ADPKD patients having a burden of biliary tract disease that can lead to rare elevation in liver enzymes. Given this patient's severe hepatic cyst burden with resulting compression of several biliary tree structures, she had an R factor (calculated as $\frac{\dfrac{\text{patient ALT}}{\text{upper limit normal ALT}}}{\dfrac{\text{patient ALP}}{\text{upper limit normal ALP}}}$) of 0.6, demonstrating a cholestatic pattern of liver injury that required intervention.

While hepatic cysts are the most common extrarenal manifestation of ADPKD, they are usually asymptomatic and incidentally found on imaging, with sequelae such as mass effect, infections, and portal hypertension rarely noted in the literature [4,7,8]. Although studies have noted biliary dilation, such as Caroli Disease, infections, cholelithiasis requiring ERCP, and portal hypertension as downstream results of PLD, it is rare to see compression of the porta hepatis alongside the presence of biliary strictures requiring stenting in this disease [2,6,8].

This raises the question of how physicians can assess patients with ADPKD for hepatic cyst burden to ensure it does not progress to this point. To assess for hepatic involvement in a patient with ADPKD, ultrasound is often used as an initial screening tool due to its accessibility and cost-effectiveness. It can identify the presence of hepatic cysts and assess their size and number [9]. Magnetic resonance imaging (MRI) is the preferred imaging modality. MRI is highly sensitive for detecting hepatic cysts and can provide detailed information on cyst size, number, and distribution, which is crucial for evaluating the extent of liver involvement [10]. MRCP can be utilized to visualize the biliary tree and detect any abnormalities [11]. Physicians should be aware to closely monitor extrarenal manifestations of ADPKD and maintain high clinical suspicion for ADPKD-related structural etiologies of liver enzyme abnormalities in such patients.

## Conclusions

Although ADPKD is primarily known as a renal disease, its extrarenal manifestations can result in adverse downstream effects on other organs, resulting in systemic dysfunction. This case emphasizes a rare instance of a hepatic manifestation of ADPKD resulting in compression of the porta hepatis, requiring advanced endoscopy and stenting, with subsequent stent migration and resulting complications leading to the patient's decompensation and eventual passing. Clinicians should thus maintain high clinical suspicion for biliary obstruction in ADPKD patients presenting with cholestatic liver enzyme elevation. Acknowledging each physiologic system via frequent reassessment is essential in the ongoing care of patients with ADPKD.
